# Spherical and cylindrical cavity expansion models based prediction of penetration depths of concrete targets

**DOI:** 10.1371/journal.pone.0175785

**Published:** 2017-05-02

**Authors:** Xiaochao Jin, Huawei Yang, Xueling Fan, Zhihua Wang, Xuefeng Shu

**Affiliations:** 1 State Key Laboratory for Strength and Vibration of Mechanical Structures, School of Aerospace Engineering, Xi’an Jiaotong University, Xi’an, Shaanxi, China; 2 Shanxi Key Laboratory of Material Strength and Structural Impact, Taiyuan University of Technology, Taiyuan, Shanxi, China; Beihang University, CHINA

## Abstract

The cavity expansion theory is most widely used to predict the depth of penetration of concrete targets. The main purpose of this work is to clarify the differences between the spherical and cylindrical cavity expansion models and their scope of application in predicting the penetration depths of concrete targets. The factors that influence the dynamic cavity expansion process of concrete materials were first examined. Based on numerical results, the relationship between expansion pressure and velocity was established. Then the parameters in the Forrestal’s formula were fitted to have a convenient and effective prediction of the penetration depth. Results showed that both the spherical and cylindrical cavity expansion models can accurately predict the depth of penetration when the initial velocity is lower than 800 m/s. However, the prediction accuracy decreases with the increasing of the initial velocity and diameters of the projectiles. Based on our results, it can be concluded that when the initial velocity is higher than the critical velocity, the cylindrical cavity expansion model performs better than the spherical cavity expansion model in predicting the penetration depth, while when the initial velocity is lower than the critical velocity the conclusion is quite the contrary. This work provides a basic principle for selecting the spherical or cylindrical cavity expansion model to predict the penetration depth of concrete targets.

## 1. Introduction

Concrete material has long played an important role in military and civil engineering constructions. Studies on penetration into concrete targets are quite necessary, especially in defense and nuclear engineering. Penetration into concrete target is a process with large strain, high strain rate, and high pressure, in which the dynamic responses of both the projectile and target are very complicated, since concrete is a brittle material that exhibits complicated nonlinear behavior [1,2]. Thus, it is difficult to make accurate predictions of the penetration process with a single formula.

**Fig 1:**
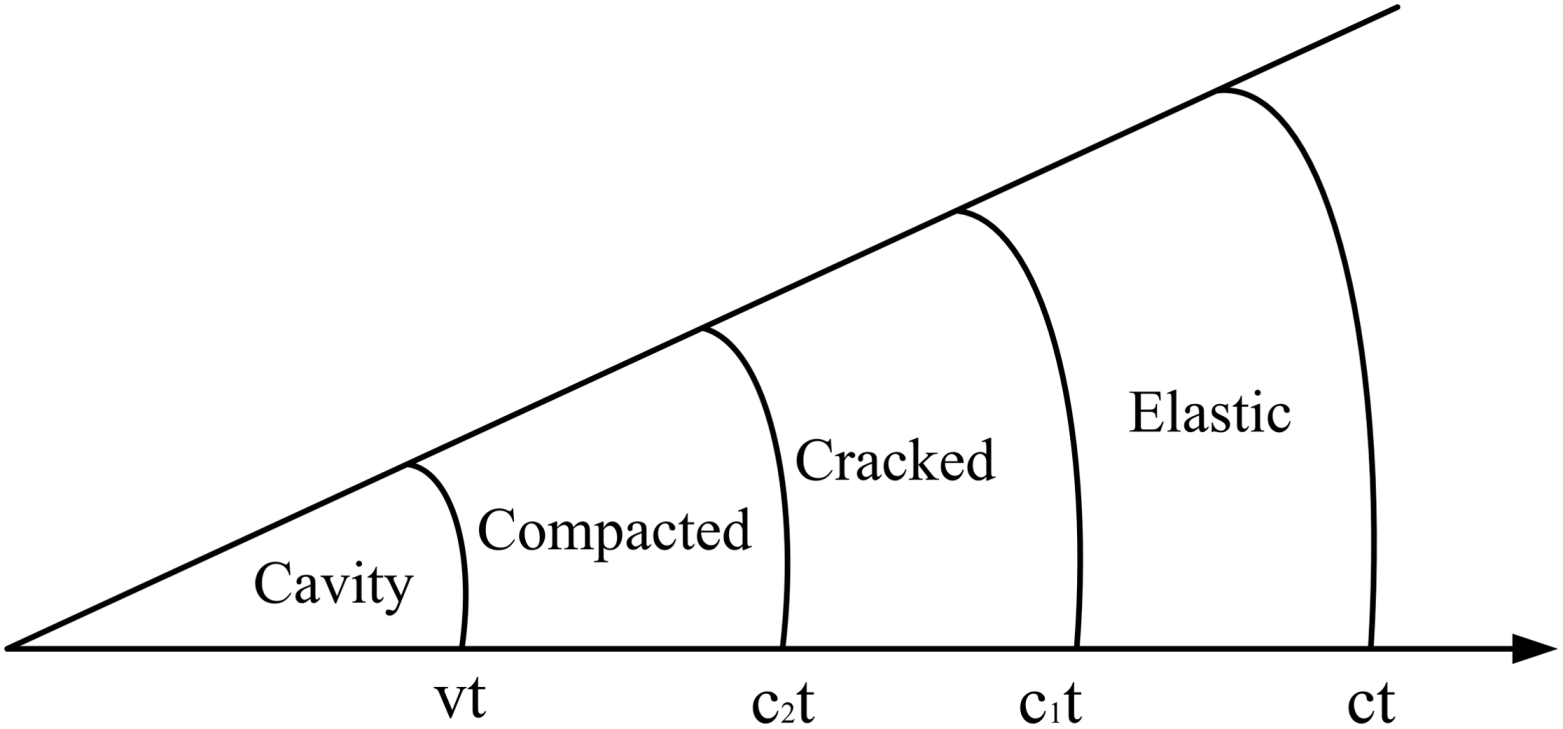
Response regions of the spherical cavity expansion model.

Over the past decades, many experimental and numerical investigations have been performed and several empirical and semi-empirical models have been developed [3,4]. Among these models, the cavity expansion theory is most widely used to predict the penetration depths of concrete targets, which was firstly proposed by Hill [5] and Bishop et al. [6]. Then, Goodier [7] proposed the theory of dynamic spherical cavity expansion and applied it to semi-infinite targets impacted by rigid spheres. Forrestal et al. [8–10] developed models for dynamic expansion of spherical and cylindrical cavities in solids by using different constitutive laws, as well as obtained approximate solutions to predict the penetration depths into metallic and concrete targets. Based on these works, Forrestal and Tzou [11] developed an elastic-cracked-plastic model for concrete targets by using spherical-cavity expansion analysis. They also compared spherical cavity expansion in concrete materials by using compressible and incompressible solid models. The results predicted through theoretical formulas approximate the experimental data. Mastilovic and Krajcinovic [12] studied the expansion of a cylindrical cavity in an infinite medium, which displays brittle behavior and inferior tensile strength. Satapathy [13] presented an elastic-cracked-comminuted dynamic cavity expansion model (Fig 1) to describe the dynamic response of brittle materials, with the assumption that the material follows a Mohr-Coulomb-type constitutive behavior. Warren et al. [14] analyzed cavity expansion in concretes through numerical simulations to assess the penetration of ogive-nosed steel rods into concrete targets. Durban and Masri [15, 16] investigated the dynamic expansion of pressurized spherical and cylindrical cavities for pressure-sensitive solids with different failure criteria: Drucker–Prager, Mises, and Tresca. Rosenberg and Dekel [17, 18] determined the critical pressure of cavity expansion in metallic targets through numerical simulations and established the relationship between pressure inside the cavity and expansion velocity for spherical and cylindrical cavity expansion. He et al. [19] and Guo et al. [20] introduced a dilatant-kinematic relation to develop spherical and cylindrical cavity expansion models, which considered the effects of shear dilatancy and compressibility, respectively.

After several decades’ research and development, Forrestal’s formula of cavity expansion theory has been the most widely used to predict the depth of penetration of concrete targets. However, as a brittle material, the effect of strain rate [21, 22] and crack propagation [23, 24] on the mechanical properties of concrete has not been clearly described, which have a significant influence on the penetration process. In addition, parameters of the cavity expansion model are difficult to accurately measure, thereby hindering the development of cavity expansion theory. Finally, both spherical and cylindrical cavity expansion theories are widely used, however, the differences between the two models in terms of predicting penetration depth have not been elucidated.

In this work, the differences and scope of application of the formulas which were used to predict the penetration depths of concrete targets based on spherical and cylindrical cavity expansion models were discussed in detail. In Section 2, penetration depth prediction based on cavity expansion theory were briefly introduced. In Section 3, numerical method were introduced and validated. Influence factors analysis and parameters fitting were conducted in Section 4. In addition, a comparative study of spherical and cylindrical cavity expansion models in predicting the penetration depth was made in Section 5.

## 2. Penetration depth prediction based on cavity expansion theory

For a compressible material, the momentum and mass conservation equations in the Eulerian coordinate of the compacted region are given as,

$$\rho (\frac{\partial \upsilon }{\partial r}+k\frac{\upsilon }{r})=-(\frac{\partial \rho }{\partial t}+\upsilon \frac{\partial \rho }{\partial r})$$ (1)

$$\frac{\partial \sigma_{r}}{\partial r}+k(\frac{\sigma_{r}-\sigma_{\theta }}{r})=-\rho (\frac{\partial \upsilon }{\partial t}+\upsilon \frac{\partial \upsilon }{\partial r})$$ (2)

where *υ* is the particle velocity measured positive outward; σ_*r*_ and σ_*θ*_ are radial and circumferential stress components, which are set as positive in compression; and *k* = 1 and *k* = 2 represent the cylindrical and spherical coordinates, respectively, which is the main difference between spherical and cylindrical cavity expansion theory.

In all the dynamic cavities, cavity expansion rate is unknown, and no thermal effects are considered. The analysis shows that radial stress on the cavity surface is a function of cavity velocity. Explicit solutions for radial stress on the cavity surface have not been established, so a numerical method have been formulated to compute radial stress on the cavity surface. Numerical results indicate that radial stress on the cavity surface can be written as [11],

$$\frac{\sigma_{r}}{Y}=A+\frac{Bv}{\sqrt{Y/\rho }}+\frac{Cv^{2}}{Y/\rho }$$ (3)

where *A*, *B*, and *C* are dimensionless and depend on the mechanical properties of solid around the cavity; *v* is the expansion velocity of the cavity surface; and *ρ* and *Y* are the initial density and yield strength, respectively. For infinite concrete targets penetrated by ogive-nosed projectiles, the equation can be simplified to

$$\sigma_{r}=S{f^{\prime }}_{c}+NB\rho v^{2}$$ (4)

$$N=\frac{8\psi -1}{24\psi^{2}}$$ (5)

where *S* is the dimensionless empirical constant, ${f^{\prime }}_{c}$ is the unconfined compressive strength of the concrete material, *ψ* is the caliber-radius-head, and *B* = 1 for concrete targets. The relationship between the expansion velocity of the cavity and the velocity of the projectile can be established by Eq (6),

v=Vcosθ (6)

Where *θ* is the angle between projectile axis and nose surface normal direction, and *V* is the impact velocity of the projectile. The applied loading is equivalent to the force exerted on the target by the projectile. For the infinite projectile nose surface *dS*, resistive force can be treated as the projection of normal force along the projectile axial direction.

$$dF=\sigma_{r}\text{cos}\theta dS$$ (7)

After integrating normal stress acting on the projectile nose, the resulting axial penetration resistive force can be expressed as

$$F=\frac{\pi d^{2}}{4}(S{f^{\prime }}_{c}+N\rho v^{2})$$ (8)

Where *d* is the diameter of the projectile. Assuming that the depth of the crater region is 2*d*, the final depth of penetration can be obtained as [9]:

$$H=\frac{2m}{\pi d^{2}\rho N}\text{ln}(1+\frac{N\rho V_{1}{}^{2}}{S{f^{\prime }}_{c}})+2d$$ (9)

Where *V*_1_ is the projectile velocity at *z* = 2*d* and is given as:

$$V_{1}{}^{2}=\frac{2mV^{2}-\pi d^{3}S{f^{\prime }}_{c}}{2m+\pi d^{3}N\rho }$$ (10)

## 3. Numerical simulations

### 3.1. Finite element model

**Fig 2:**
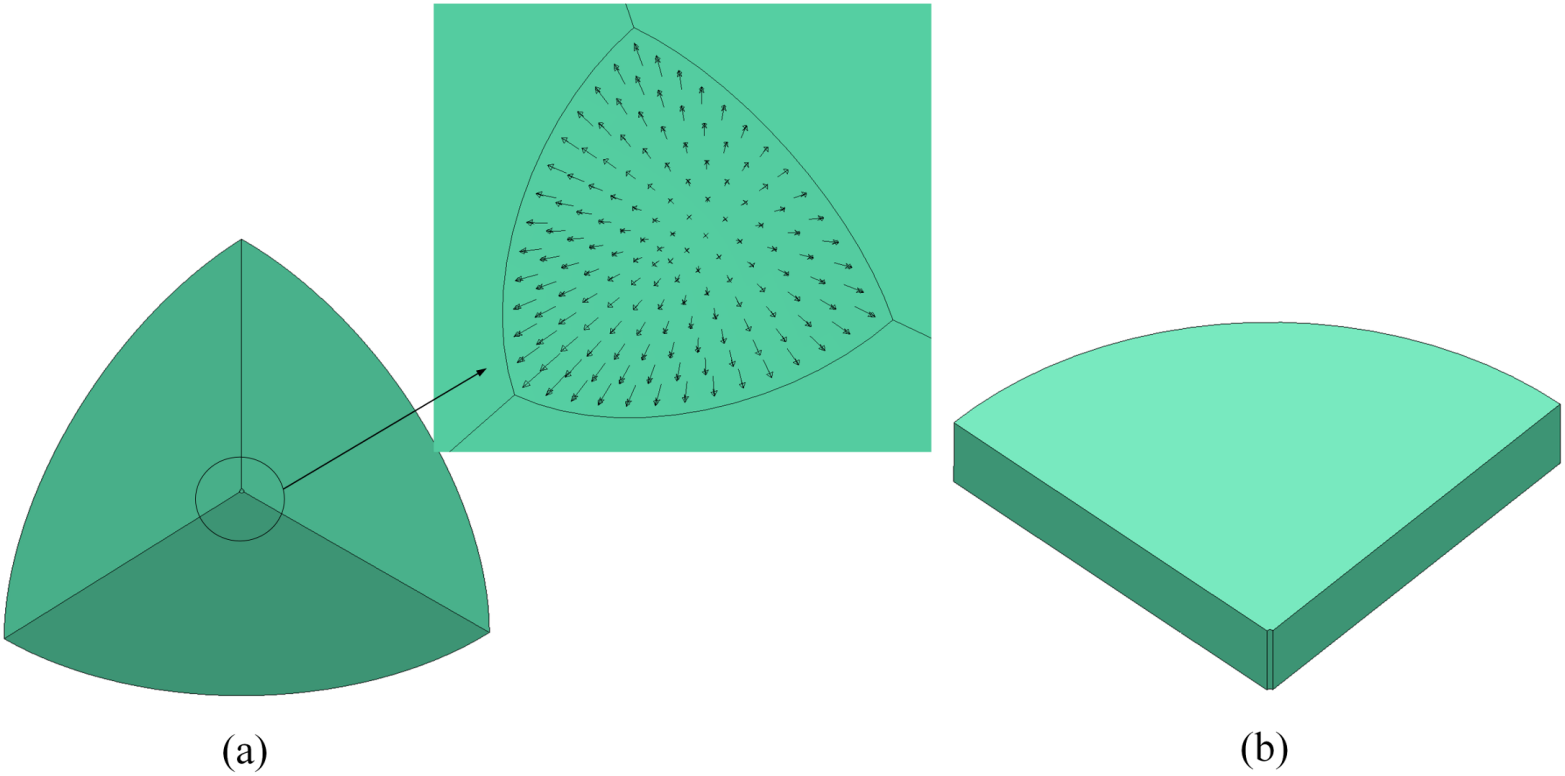
Simulation model: (a) spherical cavity and (b) cylindrical cavity.

The spherical cavity expansion model included a large sphere (200 mm diameter) with small spherical hole (2 mm diameter) in the center. Only 1/8 of the sphere was established because of the symmetry of its structure and loading. Similarly, the cylindrical cavity expansion model included a large cylinder (200 mm diameter, 20 mm height) with a small cylindrical cavity (2 mm diameter) in the center; only 1/4 of this model was established. The diameters of sphere and cylinder ensure that the compression wave will not be reflected by the free boundary within the computing time (25 μs). Thus, the concrete models can be treated as infinite. The simulation models are illustrated in Fig 2. In our simulations, the loading was applied as a boundary condition on the inner surface of the spherical cavity pre-drilled in the concrete model. The constant cavity pressure P was applied in the direction perpendicular to the surface of the cavity. In the simulation process, a Set-Segment was first created, and then a pressure-time curve was defined. After that, the defined loading curve was applied on the Set-Segment, as shown in Fig 2. Thus, the cavity expanded at the same speed along the radial direction.

The average length of elements in the region with significant deformation was 0.1 mm, whereas regions with minimal deformation were meshed with a larger cell size. The spherical and cylindrical models were meshed with 98700 and 378400 eight-node solid elements, respectively, to balance numerical convergence and computational time. Mesh sensitivity studies revealed that further refinement does not significantly improve the accuracy of the calculations but sacrifice longer of computational time.

### 3.2. Material properties

In this work, three-dimensional (3D) numerical simulations were performed using LS-DYNA [25]. The HJC model, which can be used for concretes subjected to large strain, high strain rate, and high pressure, was used for concrete sphere in the LSTC simulation. The expression is defined as

$$\sigma^{*}=\frac{\sigma }{{f^{\prime }}_{c}}=[A(1-D)+BP^{*N}][1+C\text{ln}(\overset{•}{\varepsilon })]\leq S_{\text{max}}$$ (11)

where $\sigma^{*}=\sigma /f_{c}^{\prime }$, ${\overset{•}{\varepsilon }}^{*}=\overset{•}{\varepsilon }/\overset{•}{\varepsilon_{0}}$, and $P^{*}=P/f_{c}^{\prime }$ are normalized equivalent stress, dimensionless strain rate, and normalized pressure, respectively. Here, $f_{c}^{\prime }$, ${\overset{•}{\varepsilon }}_{0}$, and S_max_ represent the quasi-static uniaxial compressive strength, reference strain rate, and normalized maximum strength, respectively. *A*, *B*, *N*, and *C*, which are material constants obtained from experimental data, represent normalized cohesive strength, normalized pressure hardening coefficient, pressure hardening exponent, and strain rate coefficient, respectively. The model incrementally accumulates damage *D* from both equivalent plastic strain and plastic volumetric strain and is therefore expressed as

$$D=\sum \frac{\Delta \varepsilon_{P}+\Delta \mu_{P}}{D_{1}{\left(P^{*}+T^{*}\right)}^{D_{2}}}$$ (12)

where Δ*ε*_*p*_ and Δ*μ*_*p*_ are the equivalent plastic strain and plastic volumetric strain, respectively; *D*_*1*_ and *D*_*2*_ are material constants; and $T^{*}=T/f_{c}^{\prime }$ is the normalized maximum tensile hydrostatic pressure. In the JHC model, the relationship between pressure and volume is defined as

$$P=\{\begin{array}{c} K_{e}\mu ,0\leq P<P_{crush}\\ P_{crush}+K_{crush}(\mu -\mu_{crush}),P_{crush}\leq P<P_{lock}\\ K_{1}\bar{\mu }+K_{2}{\bar{\mu }}^{2}+K_{3}{\bar{\mu }}^{3},P\geq P_{lock} \end{array}$$ (13)

where *K*_e,_ *K*_crush_, *K*_1_, *K*_2_, and *K*_3_ are material constants; *μ* is the volumetric strain; and $\bar{\mu }$ is the revised volumetric strain defined as $\bar{\mu }=\frac{\mu -\mu_{lock}}{1+\mu_{lock}}$. *P*_*crush*_ and *μ*_*crush*_ represent the pressure and volumetric strain, respectively; *P*_*lock*_ and *μ*_*lock*_ are the locking pressure and locking volumetric strain, respectively. In the simulations, the property parameters for concrete are shown in Table 1 (Unit: mm, mg, μs) [26].

**Table 1:** The material parameters of the 50 MPa concrete for the simulations [2,6].

**RO(mg/mm**^**3**^**)**	**G(GPa)**	**A(GPa)**	**B(GPa)**	**C(GPa)**	**N**	**FC(GPa)**
2.40	14.86	0.79	1.6	0.007	0.61	0.05
**EPS0**	**EFMIN**	**SFMAX**	**PC(GPa)**	**UC**	**PL(GPa)**	**UL**
1e-6	0.01	7	0.016	0.001	0.8	0.1
**D1**	**D2**	**K1(GPa)**	**K2(GPa)**	**K3(GPa)**	**T(GPa)**	
0.04	1.0	85	-171	208	0.005	

### 3.3. Validation of numerical method

**Fig 3:**
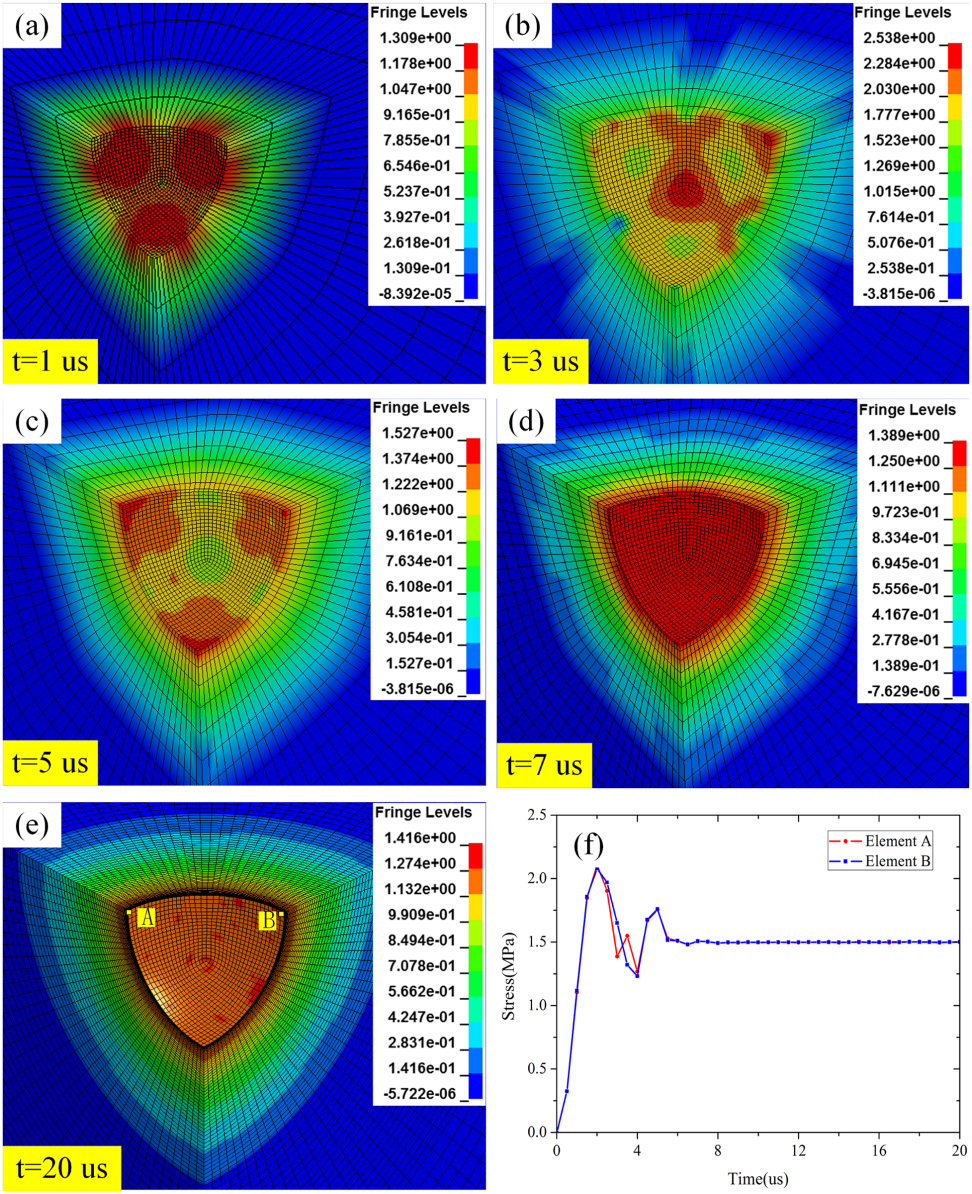
The expansion process of the spherical cavity under an expansion pressure of 1.5 GPa.

In our simulations, the loading was applied as a boundary condition on the inner surface of the spherical cavity pre-drilled in the concrete model in the direction perpendicular to the surface of the cavity. As the pressure is applied instantaneously, it is indeed a dynamic expansion process. The expansion process of the spherical cavity under an expansion pressure of 1.5 GPa is shown in Fig 3(a)–3(e), and the stress in the elements A and B on the cavity surface is illustrated in Fig 3(f). It is clear that the pressure on the cavity surface increases rapidly after a pressure was applied on the cavity surface, and the max stress on the cavity surface is higher than the expansion pressure. Then the stress on the cavity surface will decreases to a stable value at 5 us. As the aim of this work is to establish the function relationship between expansion velocity and expansion pressure, no failure model has been considered in the simulations.

**Fig 4:**
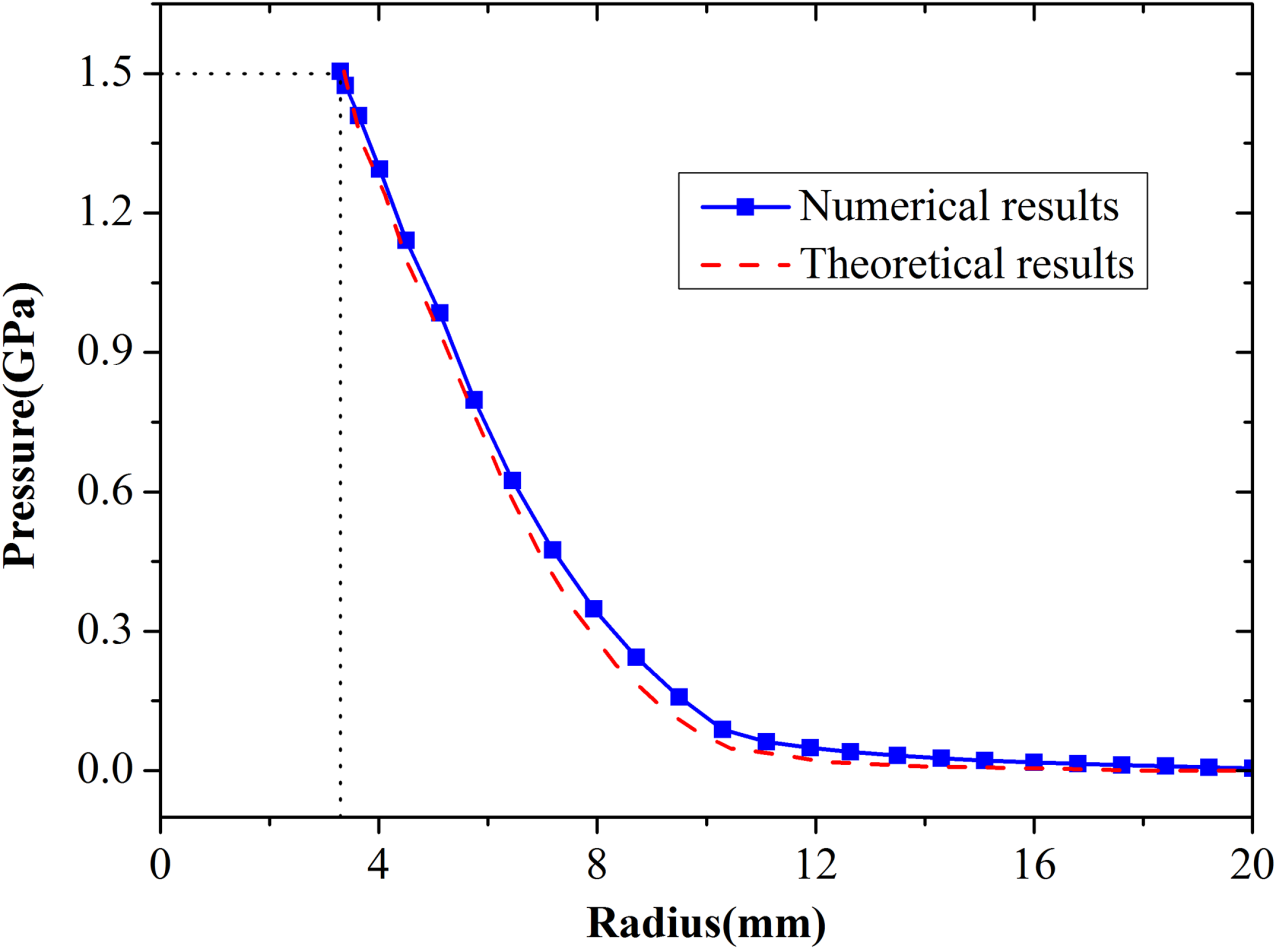
Comparison of numerical and theoretical results of radial stress after expanded for 5 us under an expansion pressure of 1.5 GPa.

The comparison of numerical and theoretical results of radial stress after expanded for 5us under an expansion pressure of 1.5 GPa was illustrated in Fig 4. At 5 μs, the outward displacement of the cavity wall reaches 2.3 mm, and the radius of the cavity reaches 3.3 mm. it is clear that radial stress values are in good agreement with the predicted values of spherical cavity expansion theory. The expansion velocity of the cavity wall increases with increasing expansion pressure, whereas the velocity of elastic compression wave remains constant. The velocity of the compacted boundary region increases with increasing expansion pressure, whereas the velocity of the cracking region boundary remains constant. Therefore, the cracked region is eventually eliminated when the expansion pressure increases. This conclusion is in accordance with the predictions of Forrestal et al. [11].

## 4. Influence factors analysis and parameters fitting

In this section, the factors that influenced the cavity expansion process were analyzed firstly. In addition, parameter fitting for spherical and cylindrical cavity expansion model were conducted.

### 4.1. Factors influencing cavity expansion velocity

As the influence factors for spherical and cylindrical cavity expansion models were the same, the analysis were only conducted based on the simulations of spherical cavity expansion model.

#### (a) Cavity pressure

**Fig 5:**
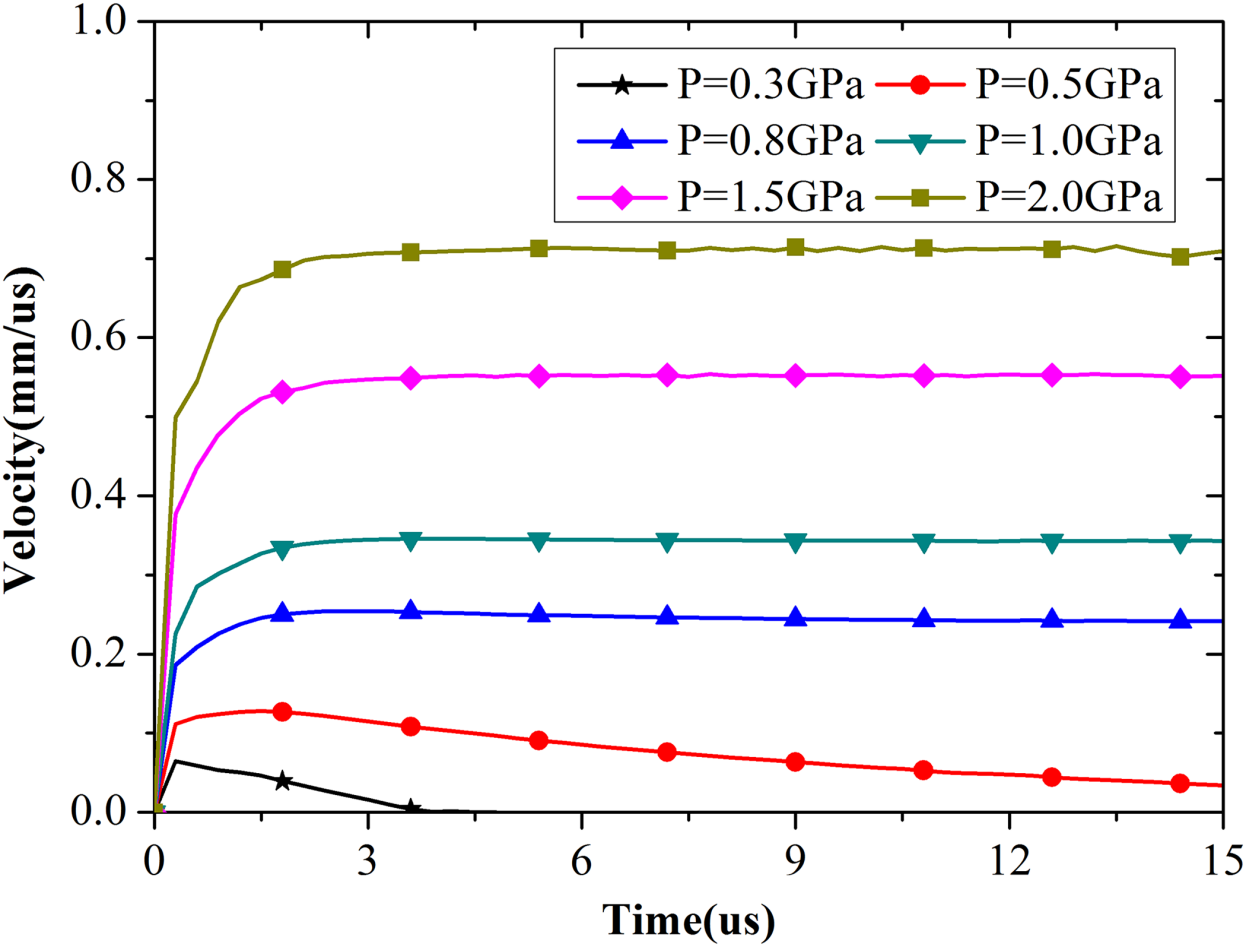
Expansion velocity of cavity wall with different pressures.

**Fig 6:**
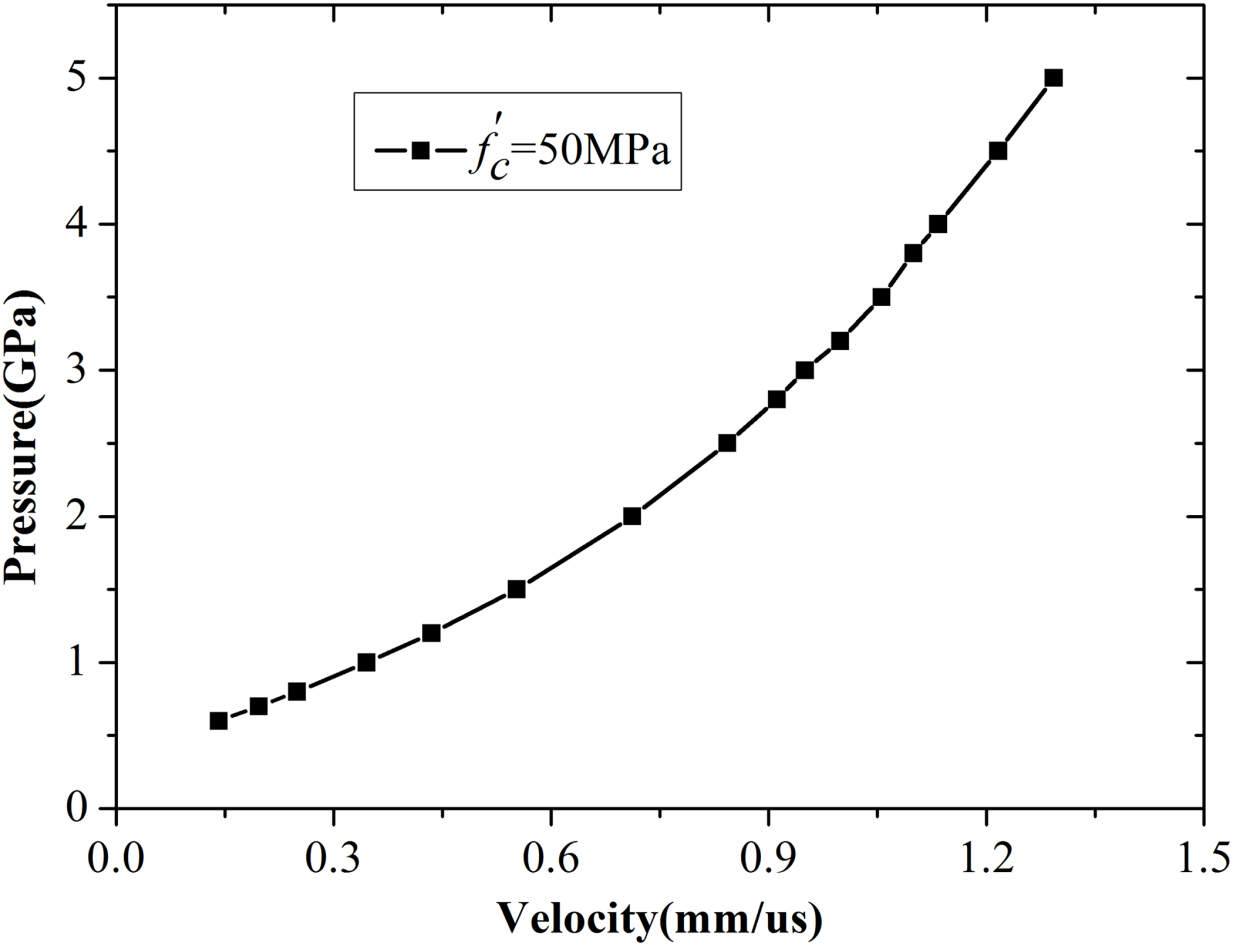
Relationship between cavity pressure and expansion velocity.

Simulations were performed with different cavity pressures, and the histories of cavity expansion velocity are shown in Fig 5. Cavity expansion velocity rapidly increases and then decreases to zero under low cavity pressures. When pressure is higher than the critical value, the velocity increases quickly and maintains a constant value. The increasing phase is longer because high cavity pressures could result in high expansion velocities. The simulation results for 50 MPa concrete are shown in Fig 6, where the nonlinear relationship between cavity pressure and expansion velocity is evident. However, expansion cannot continue when the pressure is too high because the distortion of geometry terminates the computing process.

#### (b) Initial radius of the cavity

**Fig 7:**
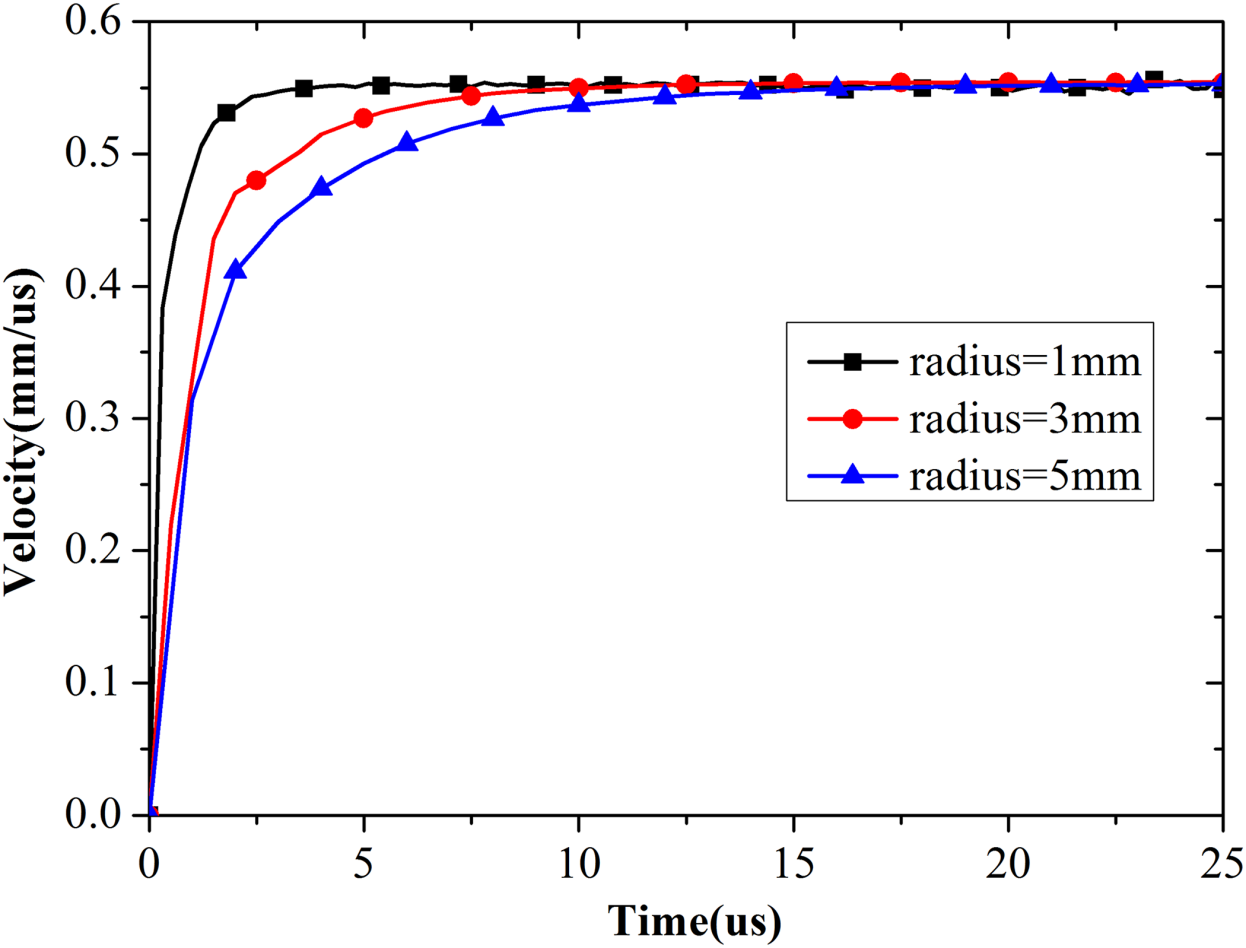
Expansion velocity of cavity wall with different initial radius.

A spherical cavity was set at the center of the concrete model to simulate dynamic spherical cavity expansion. In this section, the effect of cavity radius on expansion velocity is discussed. As shown in Fig 7, the initial radius of the cavity minimally influences the stable value of expansion velocity; by contrast, the time needed for the velocity to reach the stable phase increases with increasing initial radius of the cavity.

#### (c) Density of concrete

**Fig 8:**
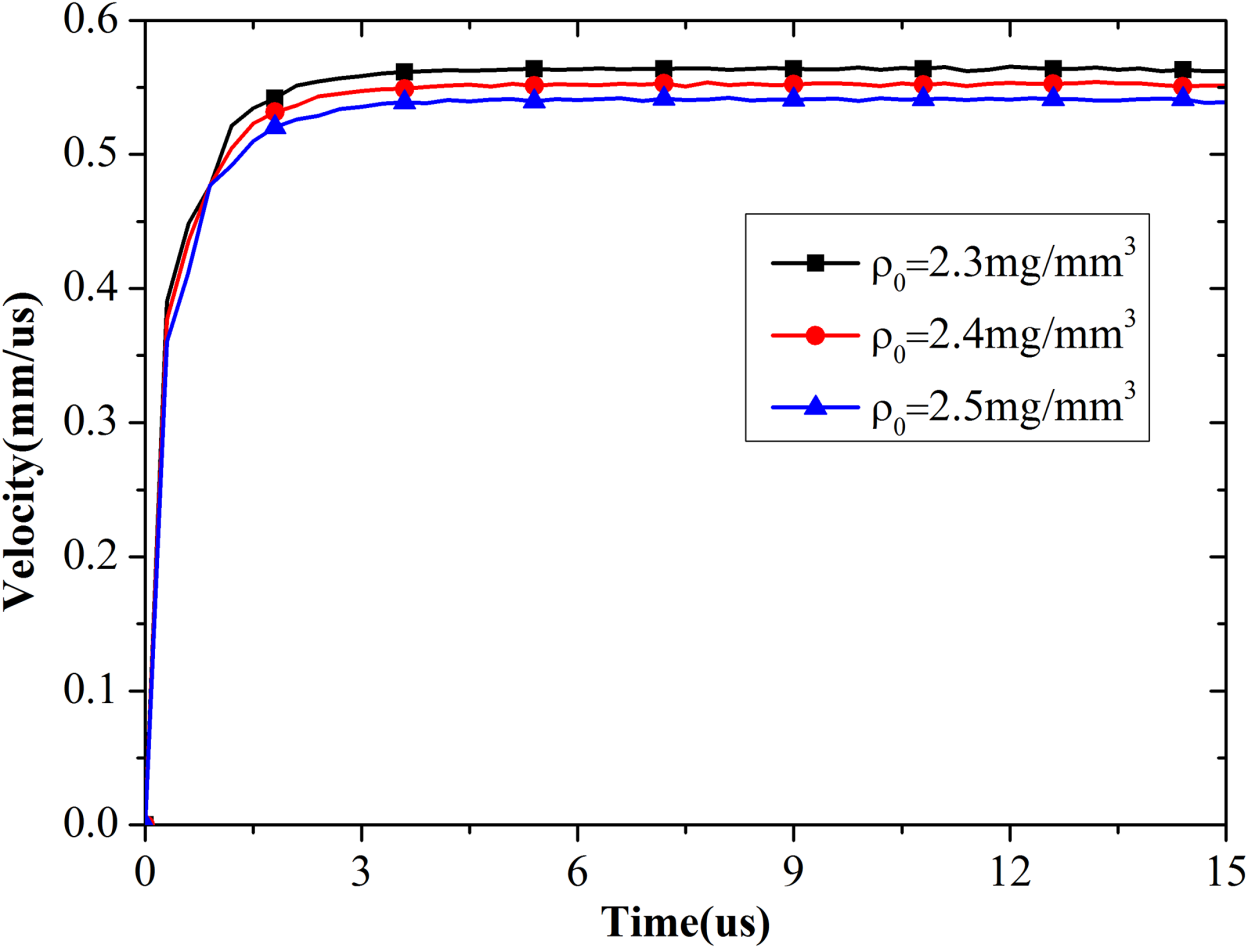
Expansion velocity of cavity wall with different densities.

**Fig 9:**
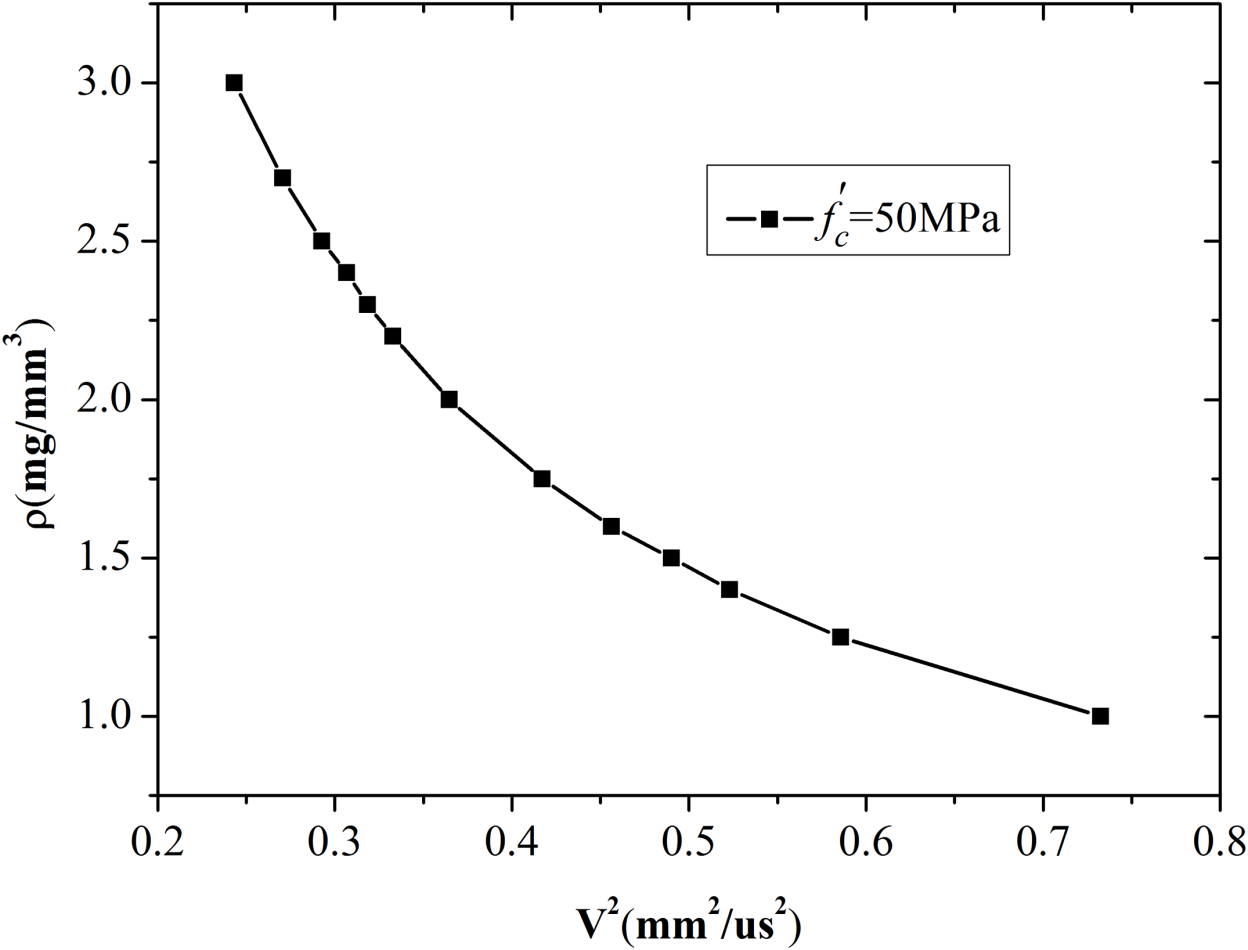
Relationship between density and square of expansion velocity.

The next set of simulations was performed for concrete with different densities to establish the relationship between density and expansion velocity. Fig 8 shows the simulation results of the expansion velocity histories. Expansion velocity decreases with increasing density. The simulation results are confirmed because high density can improve the capacity of concrete to resist deformation. Further mathematical analyses show that density is inversely proportional to the square of expansion velocity, as shown in Fig 9.

#### (d) Strength of concrete

**Fig 10:**
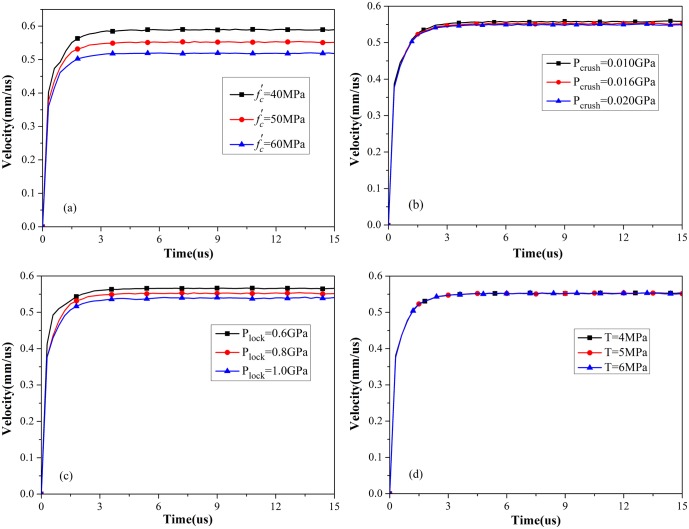
Expansion velocity of cavity wall: (a) compressive strength, (b) crushing pressure, (c) locking pressure, and (d) tensile strength.

Simulations were also performed to determine the effect of concrete strength on expansion velocity. In this section, the compressive strength, crushing pressure, locking pressure, and tensile strength are discussed. The simulation results are illustrated in Fig 10(a)–10(c). Expansion velocity decreases with increasing compressive strength, crushing pressure, and locking pressure. The influence of crushing pressure and locking pressure can be treated as the influence of compressive strength because they are dependent on compressive strength. The capability of resisting deformation is higher under high compressive strength. However, as shown in Fig 10(d), expansion velocity is independent of tensile strength. The fact that tensile strength minimally influences the expansion process is considered acceptable because cavity expansion in the simulation involves the compression of concrete and not the course of tension.

The equivalent strength of the HJC model is expressed as a function of pressure, strain rate, and damage, which accumulated as a function of the plastic volumetric strain, equivalent plastic strain, and pressure. The tensile damage of concrete materials is not considered in damage accumulation. Another set of simulations was performed with a new concrete material model called continuous surface cap model [25], which considers the tensile damage. The results are consistent with those of simulations performed with the HJC model and further confirm that tensile strength has no significant effect on cavity expansion velocity.

### 4.2. Parameter fitting for spherical cavity expansion model

**Fig 11:**
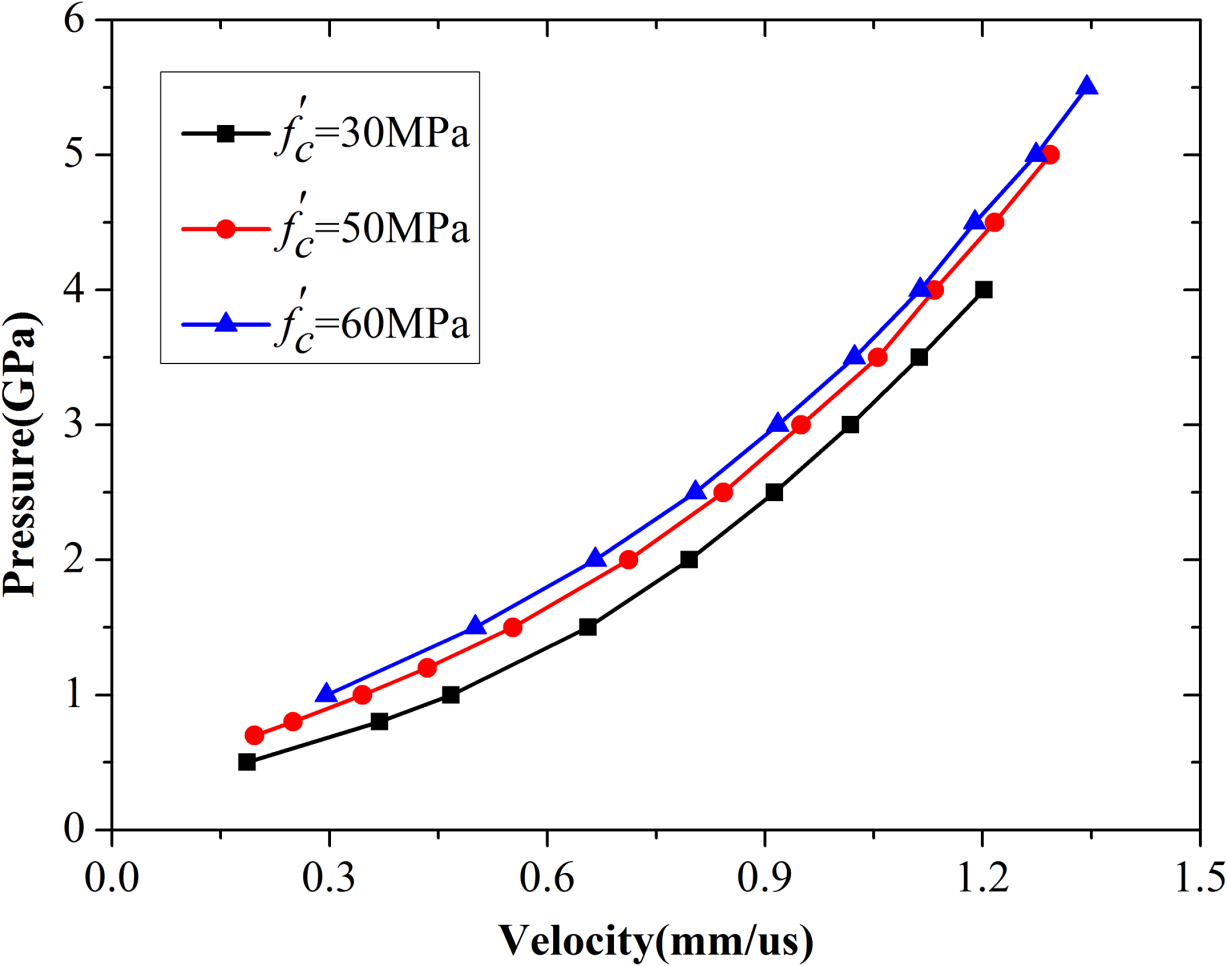
Relation between cavity pressure and expansion velocity of the cavity wall.

The expansion velocity of the cavity wall can be determined by using the pressure, compressive strength, and density of concrete. Five additional sets of simulations were performed with different compressive strengths, namely, 20, 30, 40, 60, and 70 MPa. The simulation results for 30, 50, and 60 MPa concretes are illustrated in Fig 11. Three curves exhibit similar change tendencies. A critical pressure *P*_*c*_ has been proven to ensure that cavity expansion velocity becomes constant [5, 6]. Based on the analysis of numerical data and using the polynomial fit technique, the value of critical pressures for 20, 30, 40, 50, 60, and 70 MPa concretes were designated as 0.3259, 0.4055, 0.4615, 0.5146, 0.5672, and 0.6107 GPa, respectively. The critical pressure of cavity expansion is an inherent property of concrete and is related to compressive strength. Forrestal et al. [9] proposed an empirical equation to obtain critical pressure by using abundant experimental data. With reference to the practice of Forrestal, a novel relationship between critical pressure and compressive strength was obtained based on the numerical results in the present work. The relationship can be written as Eq (14).

$$P_{c}/f_{c}^{\prime }=Af_{c}^{\prime D}$$ (14)

**Fig 12:**
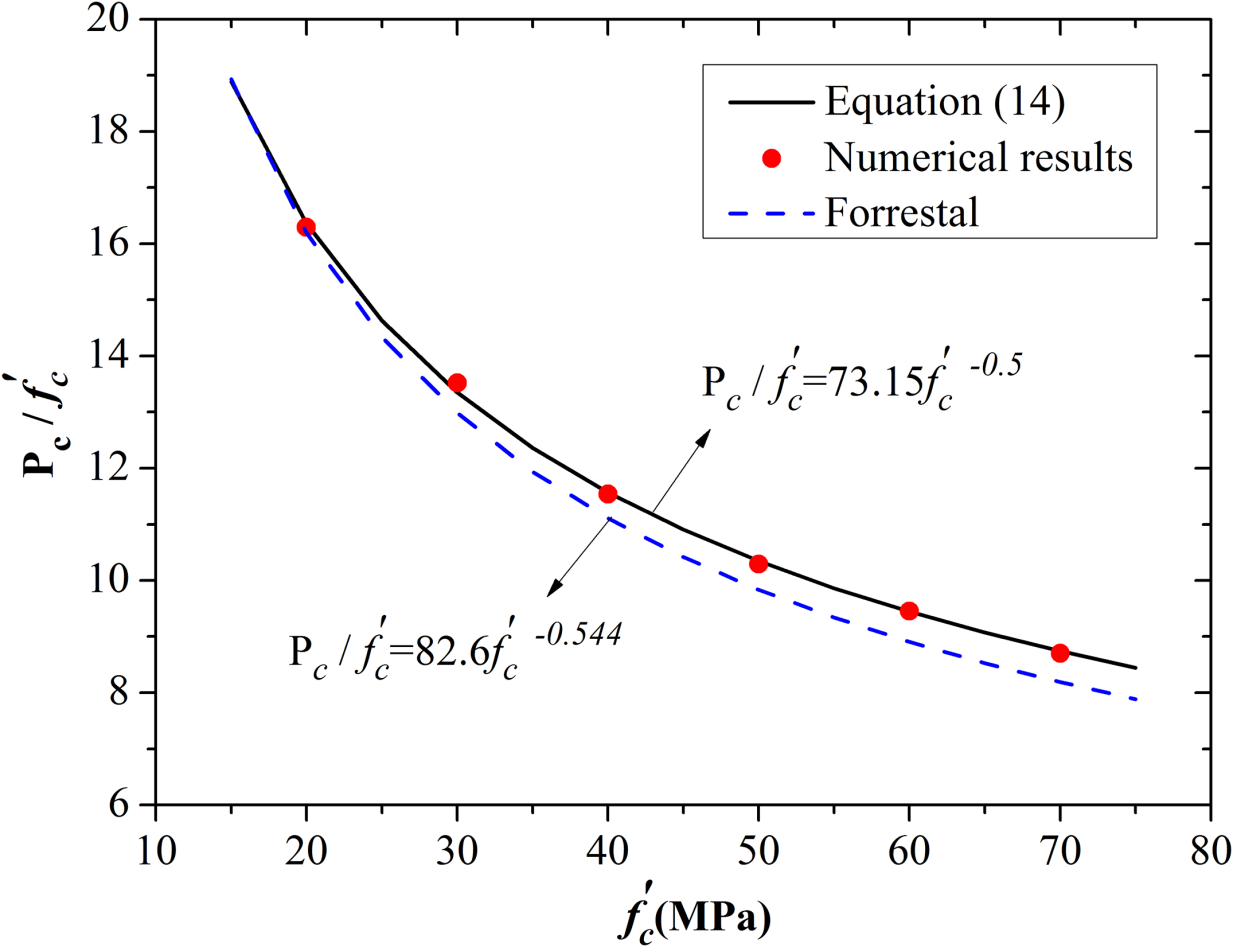
Comparison of critical pressure among best-fit curves, numerical results, and Forrestal’s formula.

Eq (14) was used to fit the six groups of critical pressure. The best-fit values of parameters *A* and *D* are 73.15 and −0.50, respectively. Comparisons of results obtained using numerical simulation, Eq (14), and empirical formula proposed by Forrestal et al. [11] are shown in Fig 12. The simulation results agree with Forrestal’s empirical fit. Similar to the procedure used by Rosenberg et al. [17], a function between expansion pressure and expansion velocity was established in terms of *P*−*P*_*c*_, as shown in Eq (15).

$$\frac{P-P_{c}}{\rho_{0}}=B^{*}V^{2}$$ (15)

**Fig 13:**
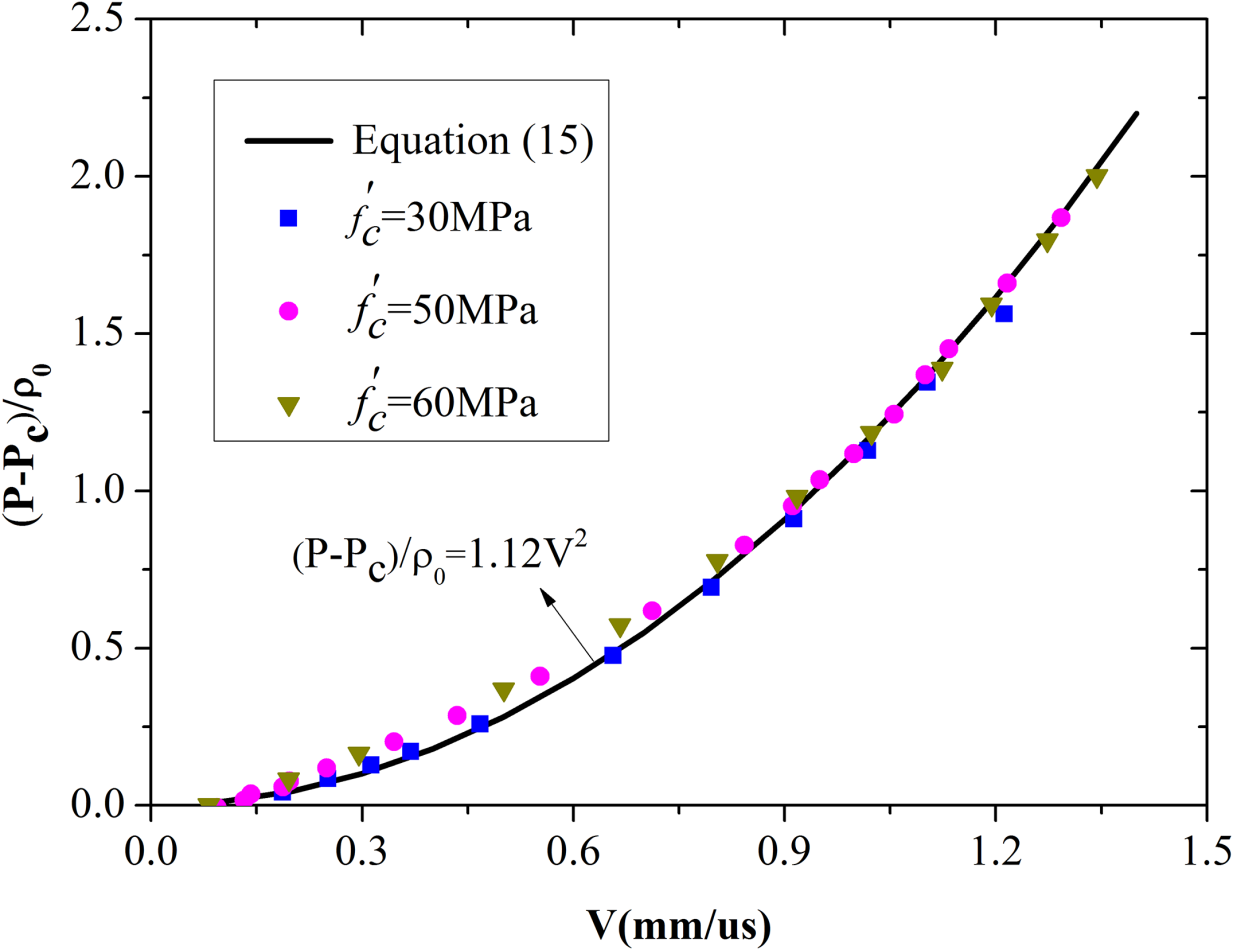
Comparison between the best-fit value of B* and the numerical results for spherical cavity expansion.

This simple quadratic equation is valid for spherical cavity expansion and can be rewritten as *P* = *P*_*c*_ + *BρV*^*2*^ [17]. A plot of numerical results along with Eq (15) can therefore be used to determine the value for the constant B*. However, the value of B* in Eq (15) is not exactly the same as that used by Forrestal et al. [9]. The best-fit value of B* is 1.12. The simulation results for 30, 50, and 60 MPa concretes and the results of Eq (15) for spherical cavity expansion are illustrated in Fig 13. The simulation results present a single quadratic curve. More importantly, the value of B* is certainly within the range of 1.0–1.2 for the spherical cavity expansion model, similar to the analytical results proposed by Rosenberg et al. [17] for different solids. Thus, the value of B* is appropriate.

After the relationship of stress and velocity was established, the penetration depths of rigid rods into concrete targets can be calculated using the function obtained in this work to determine the resistive force on the rods. Referring to the final depths of penetration *H* for a rigid projectile and a concrete target proposed by Forrestal et al. [9], a modified equation can be written using Eqs (14) and (15),

$$H=\frac{2m}{\pi d^{2}\rho NB^{*}}\text{ln}(1+\frac{NB^{*}\rho V_{1}^{2}}{P_{c}})+2d$$ (16)

Where *V*_*1*_ is the projectile velocity at *z* = 2*d* and given in the form of

$$V_{1}^{2}=\frac{2mV^{2}-\pi d^{3}P_{c}}{2m+\pi d^{3}NB^{*}\rho }$$ (17)

**Fig 14:**
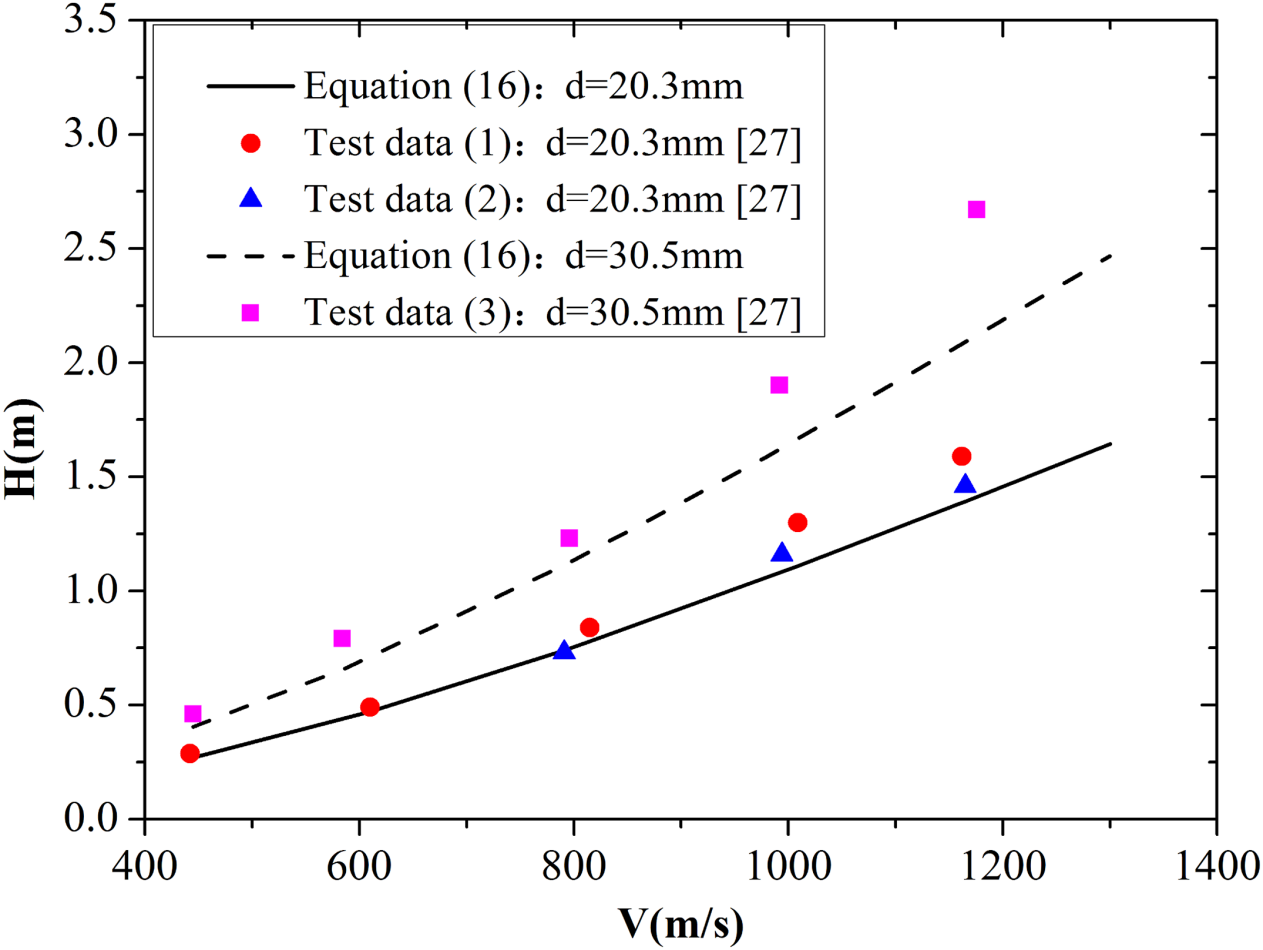
Comparison of penetration depths predicted by spherical cavity expansion model and test data.

The best-fit value of B* differs from the value of B used by Forrestal et al. [9, 11]. Frew et al. [27] provided sets of experimental data about penetration of 4340 R_c_45 and AerMet 100 R_c_53 steel rod projectiles into concrete targets, which have an unconfined compressive strength of 58.4 MPa and a density of 2320 kg/m^3^. The 4340 R_c_45 projectile exhibits 20.3 mm diameter and 0478 kg mass, whereas the AerMet 100 R_c_53 projectile possesses 30.5 mm diameter and 1.62 kg mass. Powder guns were used to launch the projectiles to striking velocities between 400 and 1200 m/s. Fig 14 shows that the experimental data are slightly higher than the values obtained from Eq (16), which can be attributed to several reasons. (1) The equations derived in this paper are based on Forrestal’s theory, in which the predicted depth of the crater region is lower than that of the experimental data, resulting in lower predicted final penetration depth. (2) According to Forrestal’s and Rosenberg’s theories, values above the critical pressure *P*_c_ should ensure that expansion velocity reaches a constant value. However, in the actual penetration, the expansion velocities of all target materials around the projectile path are not guaranteed to reach a stable value. Thus, the predicted resistance on the projectile nose is higher than the real value, which may lead to lower predicted final penetration depth. Despite the presence of errors, the calculation results are almost consistent with the experimental data. Thus, the applicability of the modified equation is verified.

### 4.3. Parameter fitting for cylindrical cavity expansion model

Similar to spherical cavity expansion, cylindrical cavity expansion was simulated using 20, 30, 40, 50, 60, and 70 MPa concretes with critical pressures of 0.2788, 0.3449, 0.4097, 0.4331, 0.5049, and 0.5334GPa, respectively. These values are about 12.34%–18.82% lower than those of spherical model. Moreover, these values approximate the prediction of the analytical model established by Bishop et al. [6], who solved the problem for incompressible spherical and cylindrical cavity expansions.

**Fig 15:**
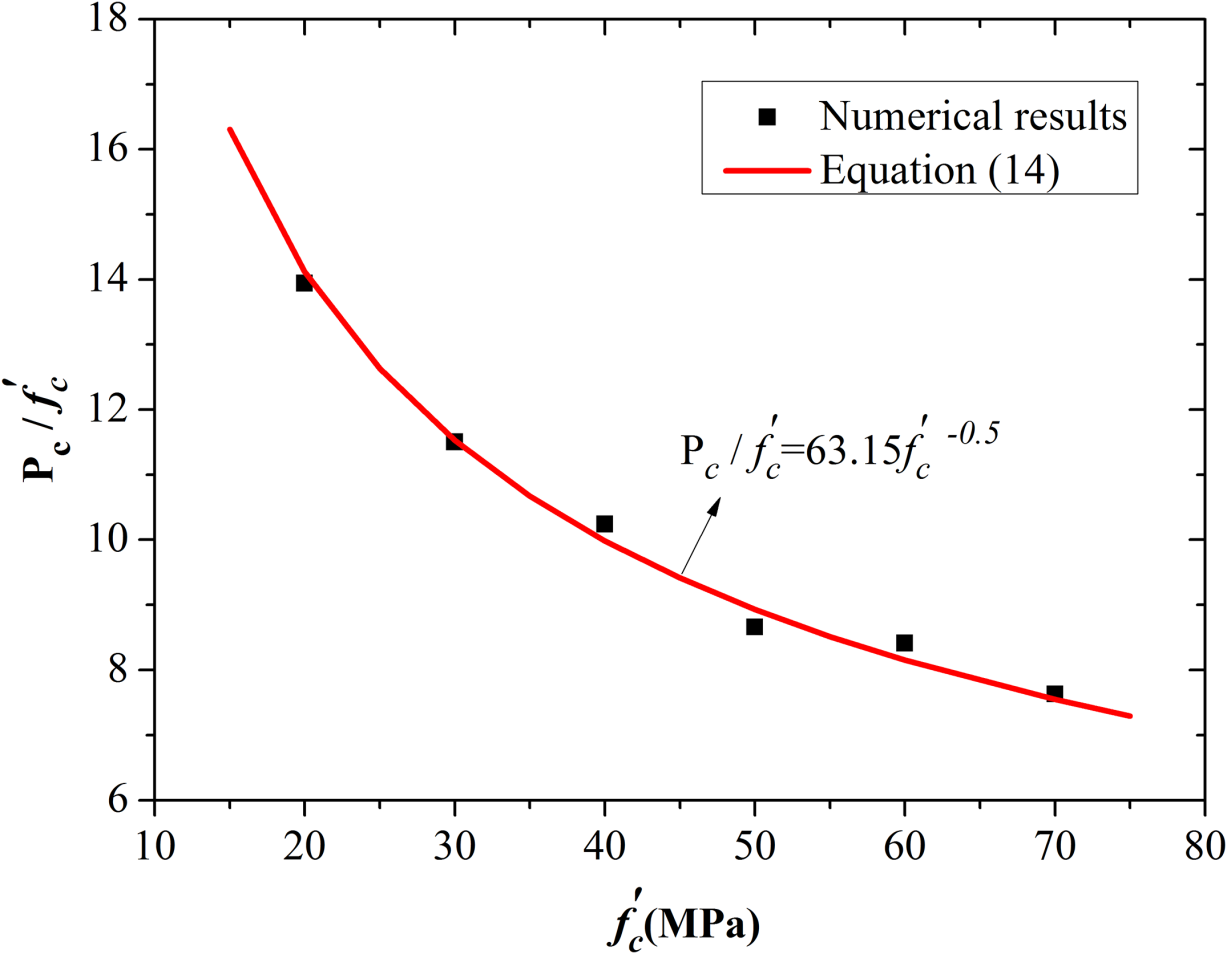
Best-fit curve of the critical pressure for cylindrical cavity expansion model.

The first issue is to establish the relationship between critical pressure and compressive strength. The same form of Eq (14) for spherical cavity expansion was adopted. The value of *D* was set as −0.5 to maintain a consistent form of the parameters. Fitting the numerical results with a nonlinear curve results *A* = 63.15. The numerical results and the fitting equation of critical expansion pressure for cylindrical cavity expansion are shown in Fig 15. The parameter value is more appropriate compared with the simulation results.

**Fig 16:**
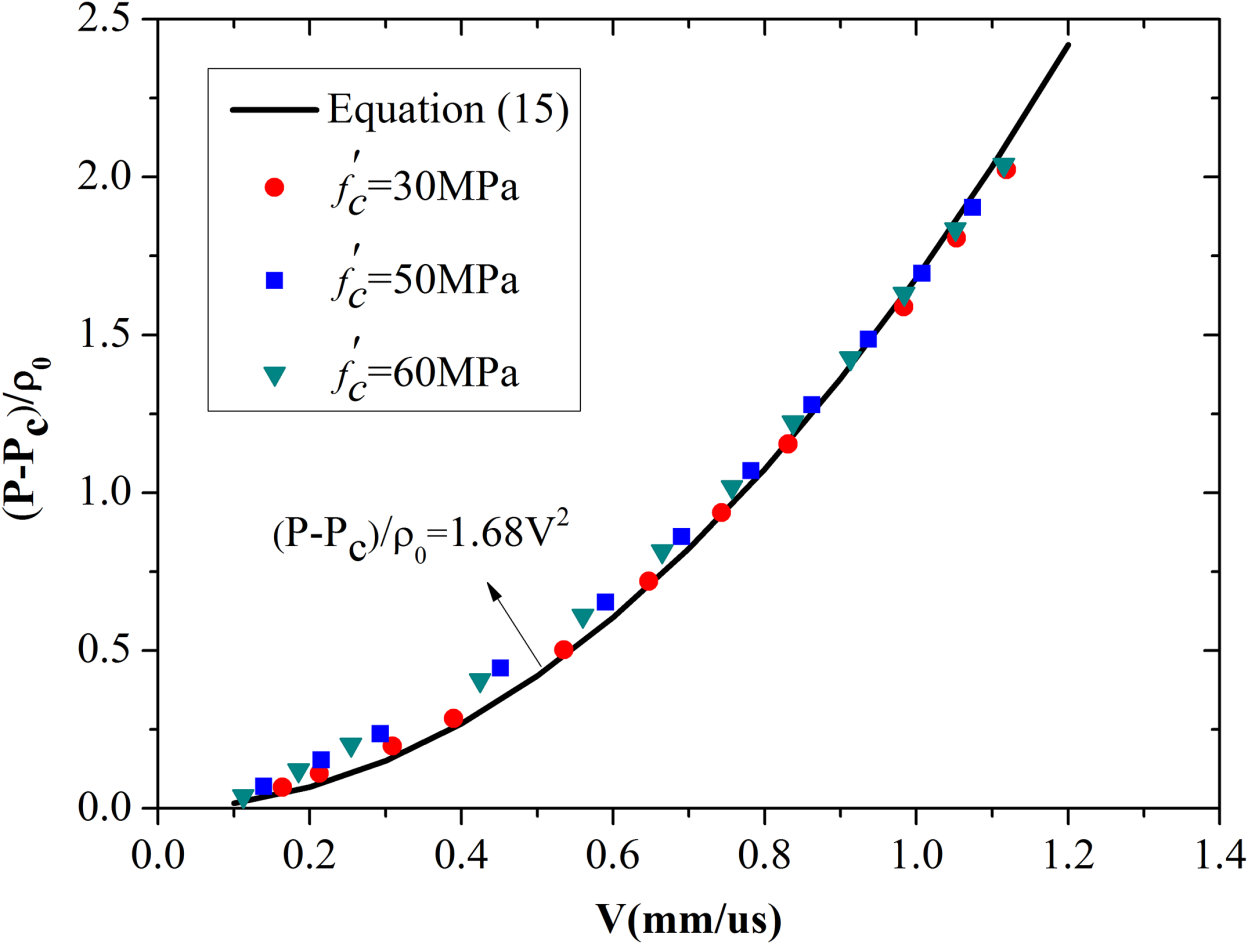
Comparison between the best-fit value of B* and the numerical results for cylindrical cavity expansion.

The next issue is concerned with establishing the relationship between expansion pressure and expansion velocity. The function took the same form as Eq (15) to simplify the calculations, but the values of parameters *A* and B* differ between the two models. The best-fit value of B* was designated as 1.68, which is higher than that of spherical model. The simulation results for 30, 50, and 60 MPa concretes and calculations with Eq (15) are illustrated in Fig 16. Almost all points for concretes with different strengths fall on the best fit curve.

## 5. Comparative study of spherical and cylindrical cavity expansion models

**Fig 17:**
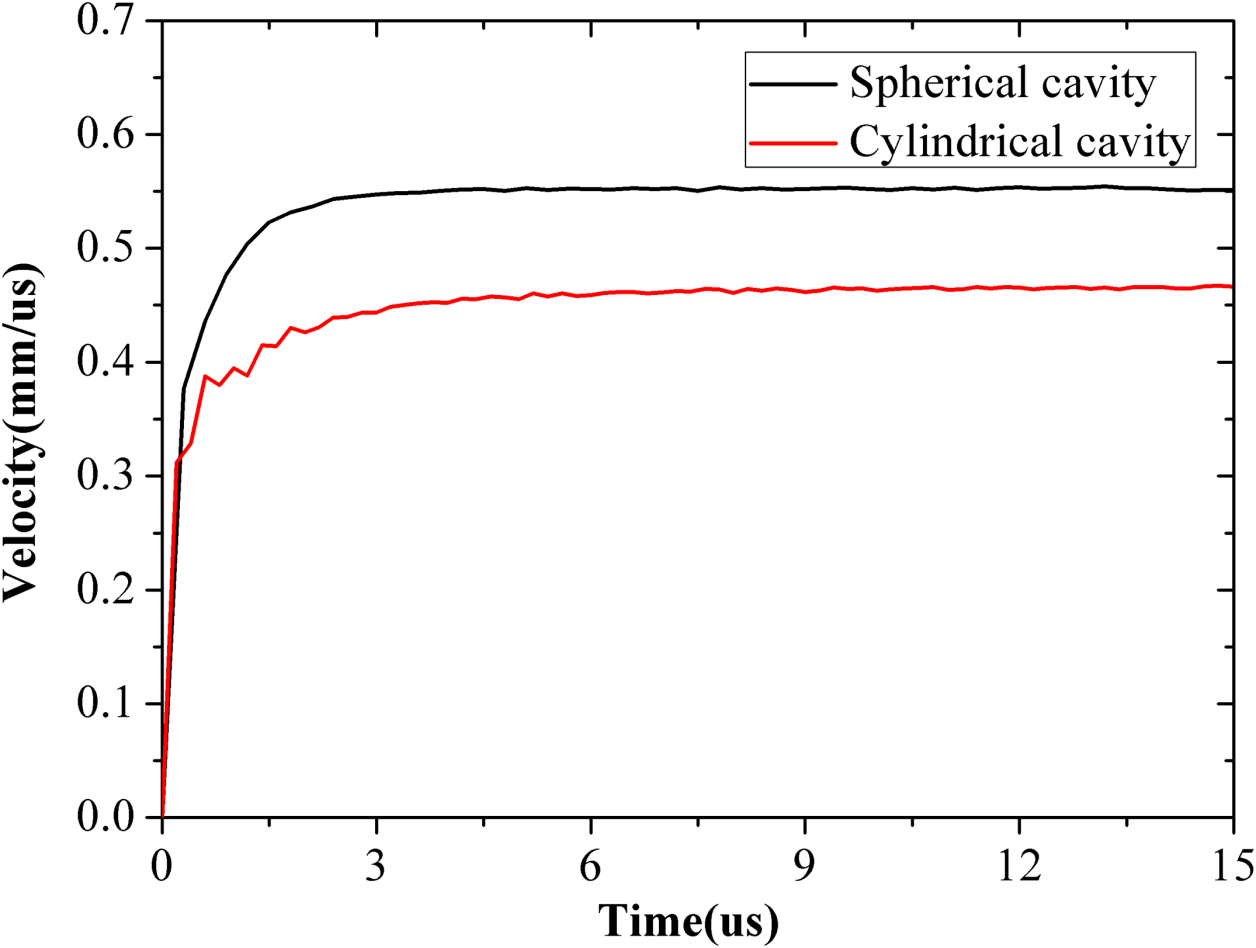
Comparison of the expansion velocity between cylindrical and spherical cavity.

**Fig 18:**
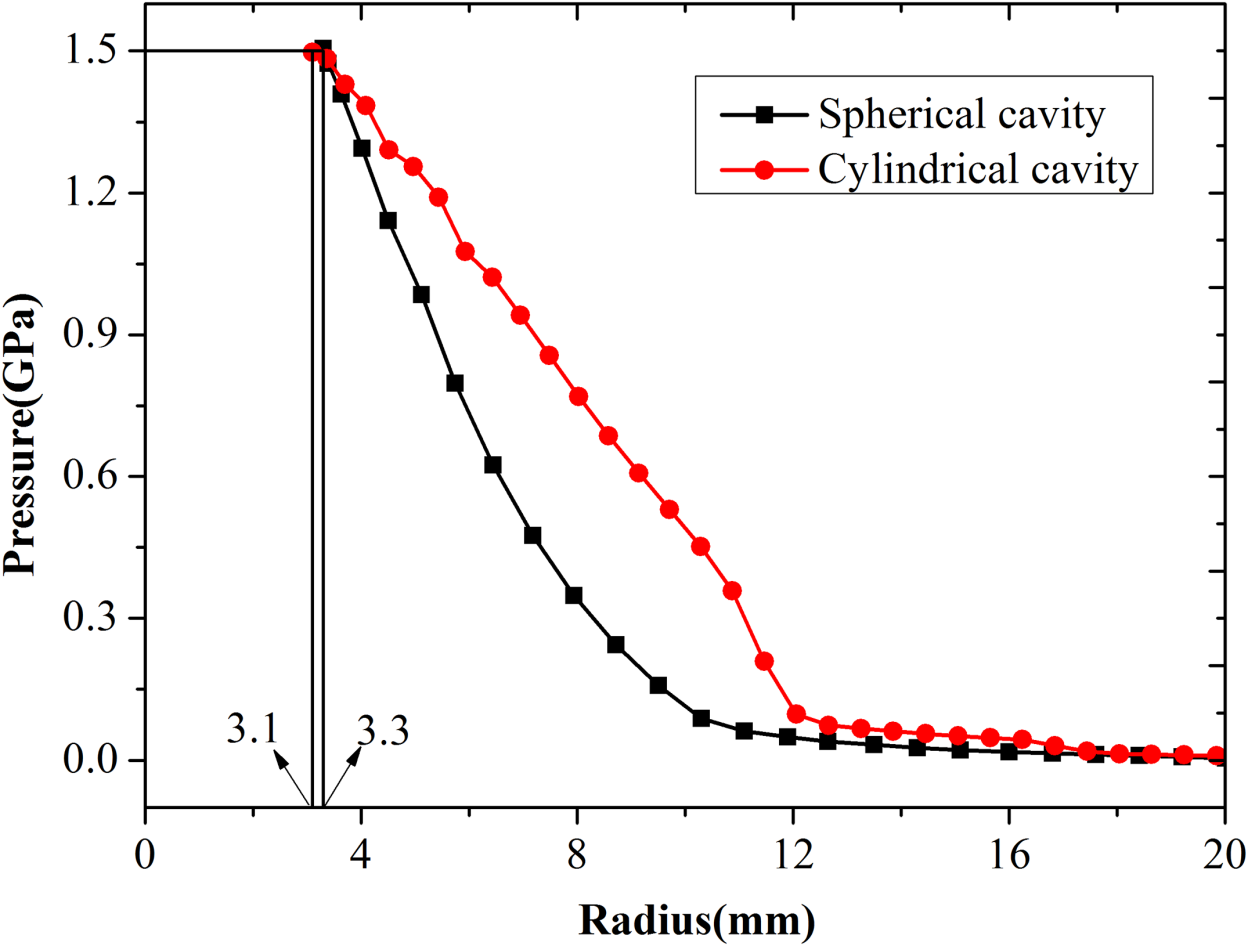
Radial stress in spherical and cylindrical cavity models at t = 5 us.

As discussed in the previous Section 4.3, the critical pressure of cylindrical cavity expansion is lower than that of spherical cavity expansion, whereas expansion velocity in the cylindrical cavity is higher under the same expansion pressure. The comparison of expansion velocity between cylindrical and spherical cavities under 1.5 GPa expansion pressure is shown in Fig 17. The expansion velocity for the cylindrical cavity (0.46 mm/μs) is about 20% lower than that of the spherical cavity (0.55 mm/μs). This finding indicates that stress at any point in the response region of the cylindrical cavity is higher than that of the spherical cavity. At 5 μs, the expansion velocities reach constant values; the outward displacement of the spherical and cylindrical cavity walls reached 2.3 and 2.1 mm, respectively; and the radius of the cavity reached 3.3 and 3.1 mm, respectively. The numerical results of the radial stress between the two models were compared, and the results are shown in Fig 18. It is obvious that the stress in the cylindrical cavity expansion model is larger than the stress in the spherical cavity expansion model with the same location.

As has been discussed, the critical pressure and velocity (under the same expansion pressure) of cylindrical cavity expansion are lower than those of spherical cavity expansion. Compared with spherical cavity expansion, cylindrical model has a lower value of *P*_*c*_ and a higher value of B*. The final penetration depths into concrete targets are dependent on both the critical pressure *P*_*c*_ (parameters *A* and *D*) and the parameter B*. The last issue highlights the differences between the two models of cavity expansion in predicting the final penetration depths of rigid projectiles into concrete targets. The penetration depths can be calculated using Eq (16) with different values of *P*_*c*_ and B* for spherical and cylindrical cavity expansion theories.

**Fig 19:**
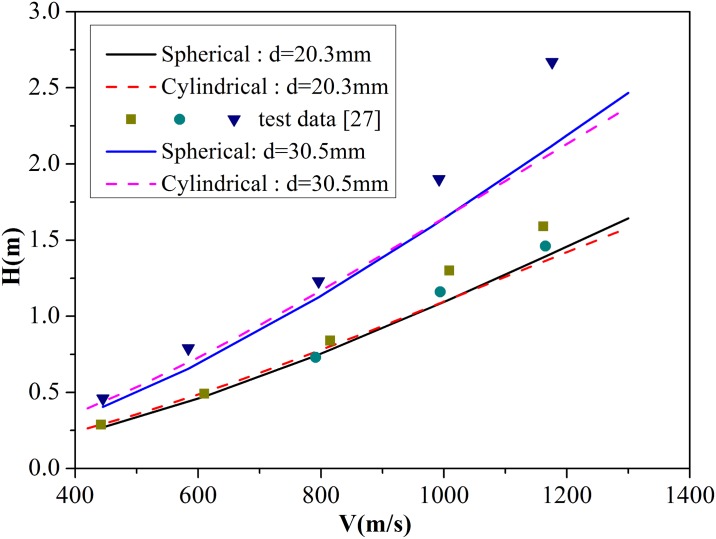
Comparison of the penetration depths obtained from cylindrical and spherical cavity expansion models with test data.

The results predicted by Eq (16) were compared with the experimental data [27] to highlight the differences and scope of application of the formulas on depths of penetration into concrete targets based on spherical and cylindrical cavity expansion models. Two groups of comparisons for projectiles with different diameters (20.3 and 30.5 mm) are shown in Fig 19. Both the formulas based on spherical and cylindrical cavity expansion models can accurately predict the depth of penetration when the initial velocity is lower than 800 m/s. However, with the increasing of the initial velocity, the prediction accuracy of formulas based on the two cavity expansion models decreases, as thermo effect on the concrete has not considered which plays an important role in high speed penetration. It can also be found that a critical velocity exists, at which penetration depths predicted by spherical and cylindrical cavity expansion models are equal. The critical velocity is determined by parameters *A*, *D*, and B*. For impact velocity lower than the critical velocity, the penetration depth from the cylindrical cavity expansion model is higher than that from the spherical model and is closer to the experimental data. By contrast, the penetration depth from the spherical model is higher and more accurate when the impact velocity is higher than the critical velocity. In addition, the prediction accuracy of formulas based on the two cavity expansion models decreases with the increasing of the diameters of the projectiles, since the formulas have not taken the size effect of the projectiles and targets into consideration. So, the formulas based on the cavity expansion models should be further developed with taking these factors into consideration in the future work. These conclusions have been validated by three groups of experimental data. Thus, this work provides a basis for selecting when to use the formula based on spherical and cylindrical cavity expansion cavity expansion to predict the depths of penetration of concrete targets.

## 6. Conclusion

In this work, the scope of application of spherical and cylindrical cavity expansion models in predicting penetration depth of concrete target was investigated. The following conclusions were drawn by analyzing the results of spherical and cylindrical cavity expansion simulations for concrete materials.

1. The expansion velocity is significantly influenced by pressure on the cavity wall, density, and unconfined compression strength of concretes, but the influence of the initial radius of the cavity and the tensile strength of concrete was insignificant.
2. The critical pressure of cylindrical cavity expansion is lower than that of spherical cavity, whereas the radial stress in the response region is higher under the same expansion pressure. Thus, a critical impact velocity exists, below which the cylindrical cavity expansion model performs better than the spherical cavity expansion model while, above which the conclusion is quite the contrary.
3. Both of the spherical and cylindrical cavity expansion models can accurately predict the depth of penetration when the initial velocity is lower than 800 m/s. However, with the increasing of the initial velocity, the prediction accuracy of formulas based on the two cavity expansion models decreases, as thermo effect on the concrete have not been taken into consideration which plays an important role in high speed penetration.
4. The prediction accuracy of formulas based on the two cavity expansion models decreases with the increasing of the diameters of the projectiles, since the size effect of the projectiles and targets were ignored in the formulas.

## Supporting information

S1 File The material model of the HJC concrete. (DOCX)

S2 File Spherical cavity expansion process. (MOV)
