# Supplementary material for: Spherical and cylindrical cavity expansion models based prediction of penetration depths of concrete targets
Source: PLoS One. 2017 May 2;12(5):e0175785. doi: 10.1371/journal.pone.0175785 (PMC5413032; doi:10.1371/journal.pone.0175785)
Supplement: S1 File — (DOCX) [file pone.0175785.s001.docx]

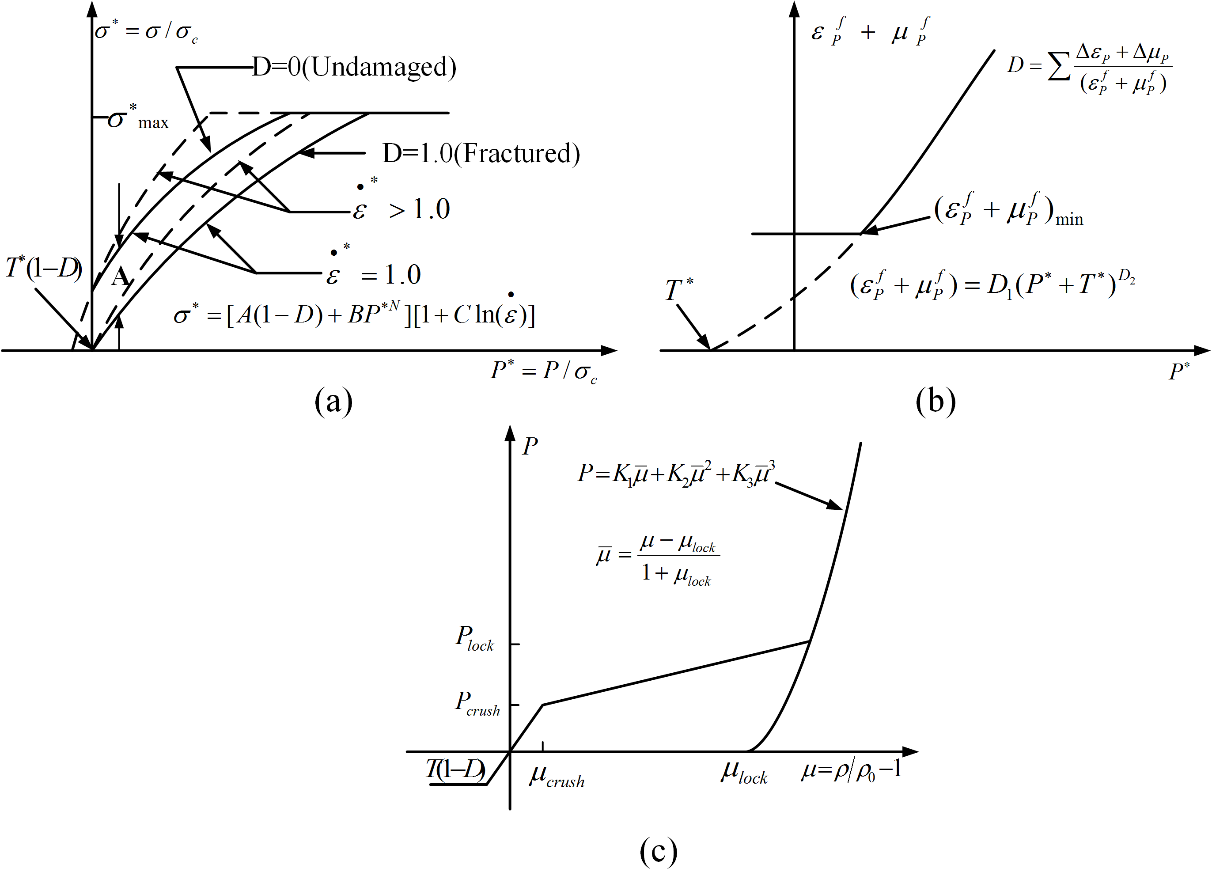


S1 File. The material model of the HJC concrete: (a) equivalent strength model; (b) damage failure model and (c) equation of state.
